# Discriminative Power of the Serious Game Attention Slackline in Children and Adolescents With and Without Attention-Deficit/Hyperactivity Disorder: Validation Study

**DOI:** 10.2196/65170

**Published:** 2025-05-13

**Authors:** Nicolás Ruiz-Robledillo, Ignacio Lucas, Rosario Ferrer-Cascales, Natalia Albaladejo-Blázquez, Javier Sanchis, Juan Trujillo

**Affiliations:** 1 Department of Health Psychology, Faculty of Health Science, University of Alicante, Alicante Institute for Health and Biomedical Research (ISABIAL), Carretera San Vicente Del Raspeig S/N, San Vicente Del Raspeig, Alicante, 03690, Spain, 34 965909900; 2 Lucentia Research Group, University of Alicante, Alicante Institute for Health and Biomedical Research (ISABIAL), San Vicente Del Raspeig, Alicante, Spain

**Keywords:** attention-deficit/hyperactivity disorder, ADHD, impulsivity, children, adolescents, neuropsychology, assessment, Attention Slackline, serious game, child, young adults, neurodevelopmental

## Abstract

**Background:**

Attention-deficit/hyperactivity disorder (ADHD) is a prevalent neurodevelopmental condition characterized by inattention, hyperactivity, and impulsivity, significantly impacting the psychological, social, and academic well-being of affected children and adolescents. Traditional ADHD diagnostic methods often rely on subjective reports, which can be biased. Recent advancements in serious games offer the potential for objective assessment tools.

**Objective:**

This study aimed to evaluate the discriminative power and concurrent validity of the serious game Attention Slackline in distinguishing children and adolescents with ADHD from those without the condition and in correlating game performance with standardized ADHD assessment scales.

**Methods:**

A sample of 32 children and adolescents diagnosed with ADHD and 39 healthy controls participated in the study. Participants were divided into 2 age groups: children (aged 6‐11 years) and adolescents (aged 12‐17 years). The serious game Attention Slackline was administered alongside established ADHD assessment scales, including the Child and Adolescent Assessment System and the ADHD Rating Scale IV. Group differences were analyzed using multivariate analysis of covariance, and effect sizes were reported using Cohen *d*. Correlations between game performance and ADHD symptoms were calculated using Pearson *r.*

**Results:**

Children with ADHD demonstrated significantly worse performance in Attention Slackline than the controls (*t*_65_=−2.26; *P*=.03; |*d*|=0.901), whereas no significant differences were observed in adolescents (*t*_65_=0.75; *P*=.73; |*d*|=0.191). Task performance was negatively correlated with family-reported hyperactivity/impulsivity symptoms in children across both tests (*r*=−0.43 and *r*=−0.51), but no significant correlations were observed in adolescents.

**Conclusions:**

The findings support the validity of Attention Slackline for assessing hyperactivity/impulsivity symptoms in children with ADHD. However, its efficacy decreases in adolescents, potentially due to developmental factors, such as compensatory strategies and ceiling effects in task performance. The gamified nature of the tool enhances engagement, which is crucial for young populations, while maintaining its diagnostic utility in measuring impulsivity. The age-dependent validity aligns with previous research indicating that continuous performance test paradigms are less effective in older populations due to developmental maturation. Attention Slackline shows potential as a complementary tool for ADHD diagnosis in children, offering an engaging and objective assessment of hyperactivity/impulsivity. Future research should aim to establish clinical cutoff points and refine the task’s complexity to align with individual characteristics.

## Introduction

Attention-deficit/hyperactivity disorder (ADHD) is defined as a neurodevelopmental disorder that must be present before the age of 12 years. It is characterized by a persistent pattern of inattention and hyperactivity, present individually or in combination, that may be maladaptive and interfere with the patient’s daily functioning [1,2]. According to data reported by the American Psychiatric Association, ADHD may be present in between 4% and 13.3% of children and adolescents worldwide, depending on the assessment protocol and the number of informants involved in the diagnostic process [2-4]. Globally, the estimated prevalence of ADHD in children is 7.6% [5]. Specifically, in Spain, a meta-analysis estimates its prevalence to be 6.8% [6]. ADHD is more frequently diagnosed in boys than in girls, reflecting its higher prevalence rate among males [7]. However, the impacts of ADHD in several dimensions regarding daily living, such as social functioning or time perception, are higher in females than in males [8].

ADHD is a prevalent disorder in the pediatric and adolescent population, characterized by significant difficulties associated with inattention, hyperactivity and impulsivity, emotional problems, and cognitive deficits [2,9,10]. This disorder represents one of the most common causes of conduct problems among children [9,11]. In this sense, children with a diagnosis of ADHD may experience a significant deterioration in psychological, social, and academic well-being [12,13]. Given its high prevalence, associated comorbidities, and chronic nature, ADHD represents a major public health concern. [14,15].

In relation to the diagnosis of ADHD, it is often necessary to rely on clinical observations, subjective reports, and objective measures of symptomatology. Clinical observations by specialists are useful but limited to the clinical environment and, thus, may not reflect the whole picture of symptoms. Assessment of ADHD using self-report ratings reveals age-related differences in perception accuracy. Children with ADHD often exhibit a positive illusory bias, overestimating their competence across various domains [16]. Conversely, adolescents with ADHD demonstrate greater awareness of their limitations, reflecting a developmental shift in self-perception [17]. Due to the difficulty of using self-reports to diagnose children with ADHD, assessment tools also consider the family or teacher’s perception of symptoms, particularly at younger ages. Therefore, it is essential to have assessment tools that allow an adequate differential diagnosis due to the subjectivity of the symptoms as reported by families and teachers, in addition to the possible overlap of diagnostic criteria of other attentional and behavioral disorders [1,14,18,19]. In this sense, neuropsychological assessment tests represent an advancement as they are specifically designed to report valid and reliable data that allow an appropriate diagnosis to be made for each patient [20,21]. Numerous studies have reported the development of standardized neuropsychological batteries that have been used in daily clinical practice in recent years and have been a turning point for the assessment and clinical diagnosis of ADHD. These neuropsychological batteries also allow for greater precision in the description of the cognitive deficits that can appear in children with ADHD and thus serve as a support for decision-making in clinical treatment [21,22]. One of the most frequently used objective paradigms for the assessment of ADHD is the continuous performance test (CPT). The CPT is a paradigm where the participant is required to respond to certain stimuli and inhibit responses to other specific stimuli. Performance in CPT has been shown to be worse in children with ADHD than their nonclinical counterparts [23]. However, this difference between them is reduced with maturation [24]. In fact, CPT has shown better discriminative power for children [25] than for adults [26].

Likewise, new technologies have been used to identify the neuropsychological profile of children with ADHD, as well as for their treatment and rehabilitation, which has shown great advantages over more traditional methodologies, reducing costs and time for diagnosis [27]. These types of tools also allow recreating different stimuli from the real world based on an ecological approach, in addition to flexibly customizing different visual and auditory stimuli in the virtual environment, such as controlling the time, order, and type of stimulus [28]. In this sense, it has been demonstrated how the use of technological measures in neuropsychological studies is valid and reliable when detecting problems in cognitive functioning [29]. A specific example of these technologies is serious games, which are defined as a type of game designed and developed with a primary purpose that goes beyond mere entertainment [30,31]. In the field of health, serious games are designed to achieve multiple objectives, including evaluation [32], diagnosis [33], and treatment of numerous pathologies [34-36]. In the context of diagnosis and evaluation, the majority of analyzed serious games have exhibited high levels of accuracy, comparable to the established classical methods [32,37,38], with even higher accuracy in some cases [33]. Specifically, in the case of cognitive assessment, serious games are a promising tool, especially for the assessment and diagnosis of neurodevelopmental disorders [39]. In the particular case of ADHD, different systematic reviews have shown that serious games are effective tools for the valid and reliable evaluation of ADHD core symptoms and associated neuropsychological dysfunctions [40,41]. Furthermore, in June 2020, the US Food and Drug Administration approved the first serious game designed for the treatment of children with ADHD [42], as part of the therapeutic process. Furthermore, more recently, other serious games have been developed as auxiliary tools for the assessment and treatment of ADHD [43-45]. However, to our knowledge, there is a gap in previous research, as the effect of relevant sociodemographic variables implicated in the diagnosis of ADHD, such as age, on serious game performance has been studied very little. Furthermore, as indicated above, analysis of the correlation between children’s and their families’ own assessment of ADHD and the serious game performance is essential. Therefore, as indicated in a recent review, research in this area is at an early stage and further studies are needed [39].

In this regard, Attention Slackline is a recently developed serious game designed to assess several ADHD symptoms. It has shown promising results in the evaluation of attentional processes in children and adolescents [46]. Although the previous pilot testing study regarding the validity of Attention Slackline reported promising results, further research is needed to validate this serious game considering wider samples, other criterion variables, and different evaluation methodologies, including self- and informant-reported tests. In this respect, one of the main issues to be considered is criterion validity.

This type of validity is commonly subdivided into 2 distinct categories: concurrent and predictive validity [47]. Concurrent validity is based on the correlation between the test to be validated and another criterion when both are measured simultaneously. Conversely, predictive validity appraises the capacity of a test to predict performance, behavior, or outcomes pertinent to the construct under assessment. In this regard, predictive validity can be regarded as an indicator of the discriminative power of a tool, allowing us to ascertain the tool’s capacity to discriminate between groups based on test results, for instance, between individuals with and without a diagnosis of ADHD. In this regard, few studies have tested the use of serious games in the evaluation of ADHD symptomatology, both in clinical and nonclinical populations, analyzing the concurrent validity of the data derived from the serious game with other subjective measures. Furthermore, little is known regarding the discriminative power of serious games in the identification of individuals with and without ADHD, especially considering the influence of significant sociodemographic factors, such as the age of the participants [48].

Taking all of this into account, the main aim of this study was to assess the discriminative power of the serious game Attention Slackline (developed by our research group) in samples of children and adolescents with and without ADHD. Furthermore, this study aimed to assess the concurrent validity of the task with self-reports and familial reports of attention and impulsivity or hyperactivity symptoms. Based on the review evidence on ADHD and CPT paradigms, we hypothesized that the task performance in the serious game Attention Slackline would be worse in those individuals with an ADHD diagnosis [40,41], especially in the younger group [16,17]. Moreover, we hypothesized that there would be a correlation between reported ADHD symptomatology and task performance [40,41].

## Methods

### Participants

The study included 32 patients diagnosed with ADHD and 39 healthy controls, all aged between 6 and 17 years. Participants younger than 12 years were classified into the children group (mean age 8.97, SD 1.61 years), whereas those aged 12 years or older were classified into the adolescent group (mean age 14.05, SD 1.66 years). Consequently, the children group comprised 10 patients and 20 controls, while the adolescents group consisted of 22 patients and 19 controls. The patients with ADHD were referred from a unit specialized in neurodevelopmental disorders from the Child and Adolescent Mental Health Service of the General University Hospital Dr. Balmis of Alicante (Spain). All patients had been diagnosed with ADHD by experienced psychologists in accordance with the criteria set forth in the *DSM-5* (*Diagnostic and Statistical Manual of Mental Disorders, Fifth Edition*). This diagnosis was reached through clinical interviews with the patients, their families, and teachers, as well as through the administration of structured tests. Healthy controls were recruited via personal referrals and recommendations from participants and study team members. Individuals with any other mental condition that can impair cognitive performance, such as intellectual disability or autism spectrum disorder, were excluded from the study.

### Instruments

#### Attention Slackline

**Figure 1.**
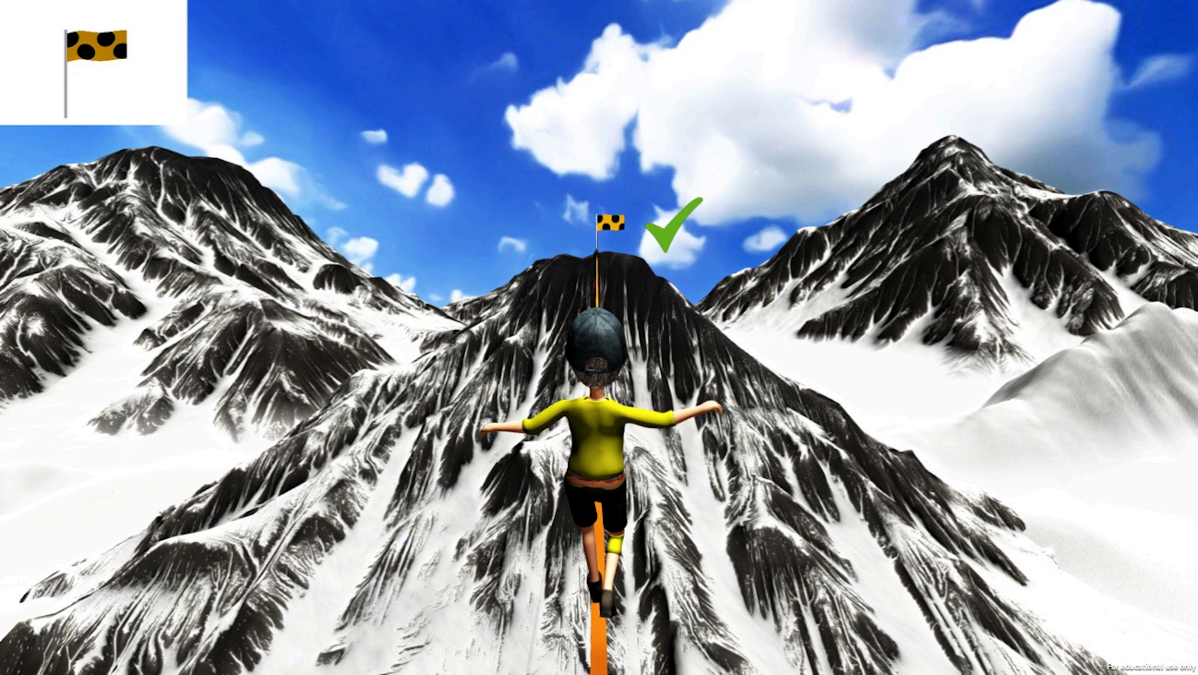
Screenshot of the Attention Slackline task.

Attention Slackline is based on a CPT paradigm [46], commonly used for assessing ADHD due to its ability to measure core symptoms such as impulsivity and inattention. The game mechanics were specifically designed to target these symptoms by requiring sustained attention and controlled responses, aligning with established neuropsychological assessment methods to ensure validity. Set in a snowy valley, the game’s narrative involves rescuing a friend isolated on a distant mountain. The player’s character, whose gender is not specified, must walk across a slackline to the opposite mountain as quickly as possible. The game is crafted to be child-friendly, with no references to hazardous situations, ensuring the character never falls from the slackline. In the Attention Slackline game, the player sees a target flag with a specific pattern in the top-left corner of the screen. Multiple flags appear on the distant mountain where the friend is located. The player’s task is to press a button when the pattern of a flag on the mountain matches the target flag. If the button is pressed correctly, the character progresses along the slackline; if not, the character slows down (Figure 1). Each game level lasts for 5 minutes and concludes with a motivational message, which is always positive regardless of the game’s outcome.

**Figure 2.**
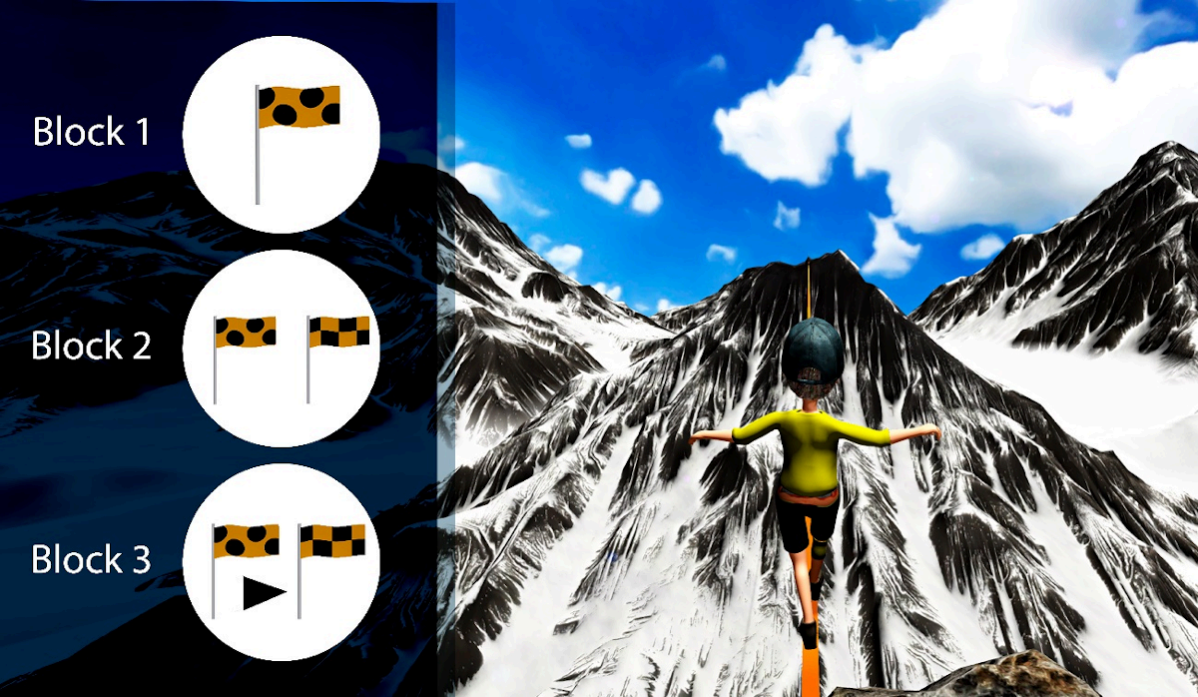
The 3 blocks of Attention Slackline used in this study.

The version of the game used in this study comprised 3 blocks of increasing complexity, as it can be observed in Figure 2. First, Block 1 introduces the basic task of pattern recognition. A target flag with a unique pattern is displayed in the top-left corner, as previously explained. Second, Block 2 increases complexity by requiring the player to track 2 target flags simultaneously. The player must respond whenever either flag appears on the mountain, testing their ability to divide attention and respond to multiple stimuli. Finally, Block 3, the final block, involves a sequential pattern recognition task, demanding higher cognitive effort. The player must identify a sequence of 2 target flags in the correct order among those on the mountain. Correct responses are registered when the player presses the button after the second flag in the sequence. Omissions are considered when the participant fails to respond to a stimulus that should have prompted a response. Commissions, on the other hand, are considered when a participant responds to an incorrect stimulus.

#### Child and Adolescent Assessment System

The attention problems and hyperactivity/impulsivity subscales of the Child and Adolescent Assessment System (SENA) [49] were applied in this study. The SENA is a well-validated instrument in Spanish adjusted by age for the assessment of emotional and behavioral problems in children and adolescents. Both the self-assessed version and the parent rating version were used.

#### ADHD Rating Scale IV

The parent’s report of the Spanish version of the ADHD Rating Scale IV (ADHD-RS) [50,51] was used for the evaluation of ADHD symptomatology. This is a well-validated instrument for the assessment of ADHD that includes inattention and hyperactivity/impulsivity subscales, as well as a combined score summing both.

### Procedure

First, the sample was selected following the inclusion and exclusion criteria described above. Professionals with expertise in ADHD from a local hospital, together with the project research team, selected the sample according to these criteria. Second, the families were contacted to explain the objectives of the project and what their participation in it would consist of. Once the families decided to participate, the informed consent form was signed by the father, mother, or the guardian of the minor. Once the informed consent was signed, participants were scheduled for 2 neuropsychological assessment sessions lasting approximately 1.5 hours. These assessment sessions took place in the University of Alicante laboratory responsible for developing this project. This laboratory consists of 4 evaluation rooms with the requirements to avoid distractions and with the appropriate sound, light, and temperature conditions to be able to carry out an evaluation of these characteristics. The neuropsychological assessment was carried out by experts in neuropsychological assessment and ADHD diagnosis. Participants were asked to abstain from taking medication before the assessment.

### Ethical Considerations

This study was approved by the ethical committee of the Alicante Institute for Health and Biomedical Research (ISABIAL; PI2022-139) and the University of Alicante (UA-2023-05-17_1). For children more than 12 years of age and less than 18 years of age, in addition to the consent of the parents or legal guardians, informed consent was also requested.

### Statistical Analysis

Statistical analyses were performed using Jamovi (version 2.3.28) for Windows. To compare age between groups, the Mann-Whitney *U* test was used, while sex differences were assessed using the chi square test. Multivariate analysis of covariance (ANCOVA) was used to evaluate differences in task performance between the clinical (ADHD) and control groups in both children and adolescents, controlling for age and sex. Task performance was calculated by using the formula 
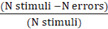
 , where errors were defined as the sum of both omissions and commissions. Normality and homogeneity of variances were checked using the Shapiro-Wilk and Levene tests, respectively. Effect sizes for the ANCOVA were calculated using partial eta squared (η^2^), with thresholds for low, moderate, and high effect sizes set at 0.06, 0.1, and 0.25 [52], respectively. Post hoc comparisons were performed using 2-tailed *t* tests to compare ADHD and control groups for both children and adolescents, with Cohen *d* used to interpret effect sizes: small (0.2), medium (0.5), and large (0.8) [53]. Pearson correlation coefficient analysis was conducted to examine the relationship between task performance and both family- and self-reported measures of attentional problems and hyperactivity. The level of confidence for all tests was set at .05.

## Results

### Age and Sex by Groups

The mean age of children in the ADHD group was comparable to that of the control group. However, there was a marginally significant difference in the number of boys and girls: 90% (9/10) of children in the ADHD group were boys, compared with 55% (11/20) in the control group (*P*=.06). The mean age of adolescents in the ADHD group was similar to that of adolescents in the control group. However, there was a difference in the number of boys and girls: 68% (15/22) of adolescents in the ADHD group were boys, compared with 26% (5/19) in the control group. (*P*<.001; Table 1). Table 2 shows the raw results of the task, divided by age and group.

**Table 1.** Sample age and sex, divided by group.

	ADHD^a^ group	Control group	*P* value
Children (n=30)			
Age (years), mean (SD)	9 (1.56)	8.95 (1.67)	.97^b^
Sex, n (%)^c^			.06^d^
Male	9 (90)	11 (55)	
Female	1 (10)	9 (45)	
Adolescents (n=41)			
Age (years), mean (SD)	14.1 (1.64)	13.9 (1.72)	.72^b^
Sex, n (%)^c^			<.001^d^^,^^e^
Male	15 (68)	5 (26)	
Female	7 (32)	14 (74)	

a ADHD: attention-deficit/hyperactivity disorder.

b Mann-Whitney *U*.

c n (%) within group.

d *χ*2.

e *P*<.05.

**Table 2.** Task results, divided by age and group.

		Mean (SD)	
Hits			
Children – ADHD^a^		51 (5.27)	
Children – Control		52.9 (5.22)	
Adolescents – ADHD		54.41 (3.05)	
Adolescents – Control		53.47 (3.79)	
Omissions			
Children – ADHD		6 (5.27)	
Children – Control		4.1 (5.22)	
Adolescents – ADHD		2.59 (3.05)	
Adolescents – Control		3.53 (3.79)	
Commissions			
Children – ADHD		19.8 (16.65)	
Children – Control		9.25 (5.84)	
Adolescents – ADHD		6.64 (5.31)	
Adolescents – Control		6.32 (4.36)	
RT^b^ hits			
Children – ADHD		1.04 (0.09)	
Children – Control		1.02 (0.1)	
Adolescents – ADHD		0.96 (0.09)	
Adolescents – Control		0.96 (0.13)	
RT commissions			
Children – ADHD		2.04 (0.62)	
Children – Control		2.01 (0.75)	
Adolescents – ADHD		2.12 (0.94)	
Adolescents – Control		2.53 (2.51)	
Task performance			
Children – ADHD		38.21 (32.62)	
Children – Control		61.32 (24.46)	
Adolescents – ADHD		72.38 (19.28)	
Adolescents – Control		70.17 (20.7)	

a ADHD: attention-deficit/hyperactivity disorder.

b RT: reaction time.

### Differences in Task Performance Between Clinical and Control Groups

**Figure 3.**
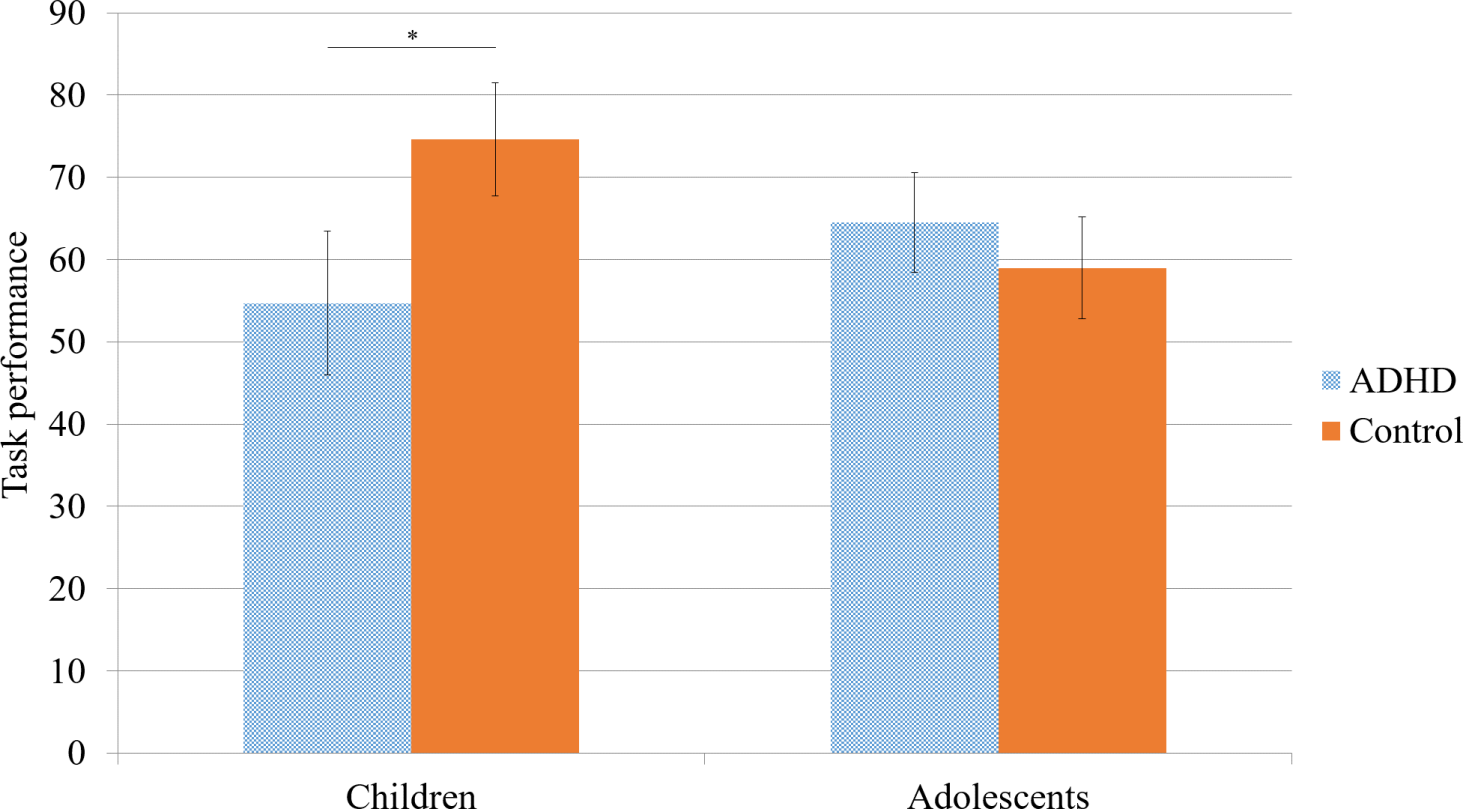
Estimated marginal means of task performance for children and adolescents with and without attention-deficit/hyperactivity disorder (ADHD). Error bars represent SE. *P<.05.

The ANCOVA analysis comparing task performance across 2 age groups (children and adolescents) with and without an ADHD diagnosis, controlling for age and sex, revealed no significant main effect between the ADHD and control groups (*F*_1,65_=1.47; *P*=.23; *η^2^_p_*=0.022). However, there was a significant group-by-age group interaction (*F*_1,65_=5.33; *P*=.02; *η^2^_p_*=0.08), indicating that the difference in task performance between the ADHD and control groups varied by age. Age itself was a significant covariate in predicting task performance, (*F*_1,65_=7.13, *P*=.01, *η^2^_p_*=0.10) while sex was not (*F*_1,65_=2.84, *P*=.10, *η^2^_p_*=0.042). The assumptions of homogeneity of variances and normality were met, confirming the validity of the analysis. Post hoc analyses showed that children with ADHD performed significantly worse than controls (*t*_65_=−2.26, *P*=.03, Cohen *d*=0.9), whereas no significant differences were found between adolescents with ADHD and their control counterparts (*t*_65_=0.75, *P*=.73, Cohen *d*=0.19; Table 3, Figure 3).

**Table 3.** Post hoc analysis of task performance differences between attention-deficit/hyperactivity disorder and control groups by age groups, using age and sex as covariates.

Age group		*t* test *(df)*	*P* value	|*d*|^a^
Children				
ADHD^b^ vs Control		−2.26 (65)	.03^c^	0.90^d^
Adolescents				
ADHD vs Control		0.75 (65)	.73	0.19

a Cohen *d* (large effect size: |*d*|>0.80).

b ADHD: attention-deficit/hyperactivity disorder.

c *P*<.05.

d |*d*|>0.8.

### Correlation Analysis

Table 4 presents the results of the Pearson correlation analysis of the relationship between task performance and self- and family-reported measures of attention and impulsivity problems for both children and adolescents. For children, there was a significant negative correlation between task performance and family-reported hyperactivity/impulsivity problems of the SENA instrument (*r*=−0.42, *P*=.02) and family-reported hyperactivity/impulsivity from the ADHD-RS (*r*=−0.51, *P*=.01). Furthermore, task performance showed a marginally significant negative correlation with family-reported combined ADHD symptoms (*r*=−0.37, *P*=.06) of the SENA. No significant correlations were observed between task performance and self-reported attention problems, self-reported hyperactivity/impulsivity, or family-reported attention problems. In the case of adolescents, none of the correlations between task performance and the measures of attention and impulsivity problems reached significance.

**Table 4.** Correlation analysis between task performance and self or family reports.

	SENA^a^ attention problems (self)	SENA hyperactivity-impulsivity (self)	SENA attention problems (family)	SENA hyperactivity-impulsivity (family)	ADHD-RS^b^ inattention (family)	ADHD-RS hyperactivity-impulsivity (family)	ADHD-RS combined (family)
Children							
*r*	−0.12	0.02	−0.26	−0.43^c^	−0.19	−0.51^c^	−0.37
*P* value	.57	.93	.18	.02^c^	.33	.01^c^	.06
Adolescents							
*r*	−0.16	−0.29	0.04	−0.03	0.09	0.16	0.14
*P* value	.33	.08	.83	.87	.60	.35	.41

a SENA: Child and Adolescents Assessment System.

b ADHD-RS: ADHD Rating Scale IV.

c *P*<.05.

## Discussion

### Principal Findings

The objective of this study was to check the validity of a newly developed serious game for the assessment of children and adolescents with ADHD [46]. The first objective was to examine the discriminative power of the serious game in groups of children and adolescents with and without ADHD. Performance on the task was significantly worse in those children with ADHD compared with their nonclinical counterparts. Nevertheless, this difference was not observed in adolescents. These results support the use of Attention Slackline for assessing ADHD in children but not in adolescents. The second objective of the study was to check the concurrent validity of the serious game with self- and family-reported attention and hyperactivity-impulsivity symptomatology. In the children’s group, the task performance was related to the measures of hyperactivity-impulsivity reported by relatives but not to attention scales or any self-reported values. Also, these results indicate that, for adolescents, task performance was not significantly associated with either self- or family-reported measures of attention and impulsivity. These findings provide support for the use of Attention Slackline as a reliable method to assess hyperactivity/impulsivity in children.

The current gold standard for ADHD assessment incorporates semistructured interviews, clinical observations, and neuropsychological tests, often complemented by teacher-parent rating scales [54]. Although CPTs are recognized as valid for diagnosing ADHD in children [55], their utility appears more limited in older populations [26]. For example, Berger et al [25] reported that children with ADHD scored significantly lower than controls on attention, timing, hyperactivity, and impulsivity indices. However, CPT performance tends to improve with age [56]. Therefore, the adolescent sample included in this study may have already developed sufficient skills to achieve a good performance in this paradigm.

Gamified interventions have shown promise for enhancing engagement and improving executive functions in children with ADHD [57]. Video game–based approaches have demonstrated utility in both assessment and treatment [40], and serious games can target the neurocognitive processes associated with neurodevelopmental disorders [39]. They also offer opportunities for customization to individual patient needs [30] and can be used to assist with both diagnosis and intervention [45]. In line with these findings, our data suggest that Attention Slackline may complement conventional methods, particularly by capturing impulsivity and hyperactivity. However, older individuals might perform near-typical levels on attention-focused tasks due to the development of compensatory strategies, consistent with concerns regarding the diagnostic power of CPTs in adults [26]. These observations highlight the importance of interpreting gamified CPT performance in the context of multiple measures and suggest that further refinements may be necessary to address subtle attentional deficits.

One of the limitations of the CPT paradigms is the ceiling effect [58,59], with a significant proportion of individuals achieving the highest score, which may be particularly prevalent in the older age groups. The paradigm used in this study was the same across all participants. As expected, task performance improved with age [56,60], with both groups of adolescents exhibiting high performance. This suggests the task may be too simple for adolescents, limiting its ability to differentiate between groups. It is possible that adolescents with ADHD may have developed compensatory strategies, which may explain the lack of correlation between task performance and ADHD symptomatology. To address this limitation, the task may be adapted by introducing context-specific distractors, such as visual or auditory stimuli, to better challenge cognitive control mechanisms in adolescents [60]. Another adaptation could involve increasing the complexity of task parameters, such as response speed requirements or number of stimuli. Increasing task complexity or tailoring its demands to developmental levels may enhance its sensitivity and applicability for this age group.

Previous CPT assessments have yielded inconclusive results with regard to parent and rating scales of ADHD symptoms [55]. The findings of the Attention Slackline paradigm in this study indicate that this paradigm is associated with the impulsivity/hyperactivity features observed in children as reported by their families. These results are congruent with the positive illusory bias, a cognitive distortion in which individuals, particularly children with ADHD, tend to overestimate their own competencies and abilities compared with objective assessments [16]. Attention Slackline appears to be a more effective method for assessing hyperactivity and impulsivity symptoms in children with ADHD than attention deficits. This could be attributed to the relatively brief duration of the task and the gamified environment. The use of a gamified environment may have augmented their engagement with the task, thereby reducing attention problems [40,45,57]. However, the use of gamified tasks would permit the observation of their inclination to respond impulsively, without fatigue, due to the duration influencing their impulsive tendency. This is why a serious game such as Attention Slackline can serve as a complement to the diagnosis of ADHD [55], serving to assess attention and impulsivity problems.

Serious games, such as Attention Slackline, are emerging as a promising addition to the clinical practice toolkit for ADHD by offering advantages over traditional assessment methods. In a narrative review, Aloisio et al [43] noted that these tools can reduce evaluation costs and time while preserving accuracy and promoting patient engagement. A randomized controlled trial by Kollins et al [42] provided further evidence by demonstrating that a digital intervention significantly reduced ADHD symptom severity in children, suggesting strong potential for real-world clinical impact. Schena et al [44] supported these findings through preliminary results showing that a virtual reality-based serious game improved core ADHD symptoms in an experimental setting, highlighting the adaptability of this approach. Zheng et al [45] additionally emphasized that serious games can facilitate both assessment and treatment, especially when integrated with large-scale data analysis. Collectively, these studies underscore the capacity of serious games to improve ADHD evaluation and intervention by offering scalable, flexible, and engaging solutions.

### Limitations

The results of this study should be interpreted in light of its limitations. First, the small size of the female population included in the sample, especially in the children’s group. Second, although the participants were instructed to abstain from taking medication before the assessment if they were under medical treatment for ADHD, possible sustained long-term effects of medication could not be controlled.

### Future Works

Future studies should aim to expand the sample size of children to determine clinically relevant cutoff points for Attention Slackline, enhancing its utility in clinical contexts [61,62]. In addition, research should explore whether a more challenging CPT could effectively distinguish between adolescents with and without ADHD. This could involve increasing the speed or duration of the task, or introducing stimuli as distractors [25]. The use of computer-gamified paradigms for ADHD assessment also opens up opportunities for incorporating machine-learning technology to evaluate performance in serious games, which may significantly improve the accuracy of ADHD diagnosis. Furthermore, future research should aim to differentiate between subtypes of ADHD [40], potentially allowing for more tailored and precise interventions based on the specific subtype diagnosed.

### Conclusions

This study provides preliminary evidence to support the use of Attention Slackline as a serious game for assessing impulsivity in children with and without ADHD diagnosis. The results suggest that the serious game is an effective tool for distinguishing children with ADHD from those without the disorder. The use of a gamified task has the potential to engage children in the task and demonstrate their impulsive tendencies in responding. Early identification of ADHD symptoms is crucial to facilitate timely and appropriate interventions. The use of a serious game such as Attention Slackline as an additional assessment tool may provide a useful objective measure of impulsivity in children.

## Abbreviations

ADHD: attention-deficit/hyperactivity disorder
ADHD-RS: ADHD Rating Scale IV
ANCOVA: analysis of covariance
CPT: continuous performance test
*DSM-5*: *Diagnostic and Statistical Manual of Mental Disorders, Fifth Edition*
SENA: Child and Adolescent Assessment System (Sistema de Evaluación de Niños y Adolescentes)
